# Utility of a Specific Health Checkup Database Containing Lifestyle Behaviors and Lifestyle Diseases for Employee Health Insurance in Japan

**DOI:** 10.2188/jea.JE20180192

**Published:** 2020-02-05

**Authors:** Toshiki Fukasawa, Nanae Tanemura, Shinya Kimura, Hisashi Urushihara

**Affiliations:** 1 Division of Drug Development and Regulatory Science, Graduate School of Pharmaceutical Sciences, Keio University, Tokyo, Japan; 2 Japan Medical Data Center Co., Ltd., Tokyo, Japan

**Keywords:** health checkup, lifestyle behavior, lifestyle disease, national survey, Japan

## Abstract

**Background:**

The Japanese Ministry of Health, Labour and Welfare introduced Specific Health Checkups (SHC) to identify individuals at risk of metabolic syndrome (MS). This study aimed to describe the SHC database developed by the Japan Medical Data Center Co., Ltd. (JMDC) as a means of exploring lifestyle behaviors and lifestyle diseases among working generations.

**Methods:**

We conducted a retrospective, cross-sectional study of employees and their families using the JMDC-SHC database to describe the prevalence of lifestyle behaviors (smoking, exercise, dietary habits, drinking habits, and sleeping) and lifestyle diseases (MS, hypertension, dyslipidemia, and diabetes mellitus). Results were compared with data from the 2015 National Health and Nutrition Survey (NHNS) in Japan as a benchmark.

**Results:**

All 646,869 enrollees in the JMDC-SHC database were included, of whom 66.5% were men. Age ranged from 40–74 years. Compared with the results of the NHNS, the JMDC-SHC subjects were younger and had fewer MS components and a lower prevalence of diabetes and hypertension. Subjects in their 40s were most likely to have unhealthy lifestyle behaviors in all age groups (eg, smoking: 41.0% in men and 10.2% in women). The SHC group had more favorable behaviors overall, but underweight was more prevalent in the SHC females.

**Conclusions:**

The JMDC-SHC population showed different lifestyle and lifestyle disease profiles to the NHNS population, probably due to its different age, gender, and employment distributions. Development of healthcare policies and plans for working generations would benefit from the selection of an age- and employment-appropriate database.

## INTRODUCTION

In 2008, the Japanese Ministry of Health, Labour and Welfare (MHLW) introduced annual health screening and health promotion services, called “Specific Health Checkups (SHC) and Specific Health Guidance (SHG),” as part of the national health insurance system.^1^ The SHC is aimed at early detection and intervention for people with or at high risk of metabolic syndrome (MS), a common lifestyle-related disease. Insured persons and their dependents aged 40–74 years are interviewed by industrial doctors, take a questionnaire survey of lifestyle, and undergo lab tests for metabolic diseases, including hypertension, dyslipidemia, and diabetes mellitus. Individuals suspected of having lifestyle diseases at the SHC are monitored by the employer and strongly recommended to seek medical advice or intervention to prevent lifestyle diseases, improve lifestyle behaviors, and promote self-health management. Additionally, the 2013 Japan Revitalization Strategy of the Japanese government required all health insurance societies to develop health promotion plans, called “Data Health Plans,” to maintain and improve the health status of their subscribers and reduce medical costs by analyzing health data, including SHC data and claims data.^2^

Some large-scale business enterprises run healthcare insurance plans that provide healthcare insurance coverage and healthcare management to their employees. This type of health insurance plan accounts for about 24% of all health insurer plans in Japan.^3^ The Japan Medical Data Center Co., Ltd. (JMDC) has provided a large-scale claims database derived from some of these employees’ insurers. Since 2008, this has been linked with the SHC data (SHC database). The population covered by the SHC database consists of more than six hundred thousand covered individuals including employees and their dependents, and accounts for about 5.4% of subscribers of all health insurance societies.^4^ The consumption of healthcare resources and the results of annual SHC of the covered individuals can be tracked in the database, unless they withdraw from their health insurance society. The enrollees of the JMDC-SHC database are employees of large enterprises from the secondary and tertiary industries and their dependents.

In this study, we summarized the prevalence of lifestyle behaviors and lifestyle diseases among people in the SHC database provided by the JMDC in comparison with data from the National Health and Nutrition Survey (NHNS) as a benchmark to characterize the JMDC-SHC database.^5^ Our goal was to evaluate the utility of the JMDC-SHC database for epidemiological studies aimed at enhancing and promoting healthcare management in workers.

## METHODS

### Design and data source

The study was conducted under a retrospective, cross-sectional design using the JMDC-SHC database (April 1, 2015 through March 31, 2016). The JMDC-SHC database contained the data of employees and their dependents aged 40–74 years enrolled in health insurance plans that were run mainly by large-scale enterprises. All enrollees in the JMDC-SHC database were eligible for participation in the study.

The SHC records general information (sex, date of birth, and date of SHC), anthropometric measurements (height, weight, body mass index [BMI], waist circumference [WC]), laboratory values (systolic blood pressure [SBP], diastolic blood pressure [DBP], triglyceride, high-density lipoprotein cholesterol [HDL-C], low-density lipoprotein cholesterol [LDL-C], aspartate aminotransferase [AST], alanine aminotransferase [ALT], γ-glutamyl transpeptidase [γ-GTP], fasting blood glucose, sugar in the urine, protein in the urine, and hemoglobin A1c [HbA1c]). The SHC is also used to record the results of a self-administered questionnaire on lifestyle behaviors (current smoking, regular exercise, dietary habits, drinking habits, and sleep habits) and medications for hypertension, dyslipidemia, and diabetes mellitus.^1^ The JMDC-SHC database includes all of the above data.

Summary level results of the NHNS in 2015 are publicly available for comparison with the JMDC-SHC database. The NHNS is an annual nationwide statutory statistical evaluation conducted by the MHLW under the Health Promotion Act which surveys the health status, nutritional intake, and lifestyle behaviors of Japanese nationals.^5^ Japanese residents aged ≥1 year living in 300 census units are surveyed as part of the Comprehensive Survey of Living Conditions.^6^ The NHNS shares some common survey items with the SHC.

The study protocol and waiver of informed consent were approved by the Ethics Committee for Research Involving Humans of Keio University Faculty of Pharmacy, Tokyo, Japan (No. 160909-2), in accordance with local ethical guidance for medical research involving human subjects.^7^

### Variables

We extracted the following data from the JMDC-SHC database: general information (sex, date of birth, and date of SHC), anthropometric measurements (BMI and WC), laboratory values (SBP, DBP, HDL-C, and HbA1c), and the results of the self-administered questionnaire on lifestyle behaviors (current smoking, regular exercise, dietary habits, drinking habits, and sleep habits). The self-administered questionnaire items on lifestyle behavior were defined based on the definitions used for the NHNS and the annual general health checkup conducted under the Industrial Safety and Health Act (eTable 1).^1^^,^^8^ Some of these were equivalent to the survey items in the NHNS. Excessive drinking was defined by answers to the questions on drinking habits of both “occasionally or everyday” and “≥180 mL of sake (equivalent to ≥ 20 grams of alcohol)”. Subjects who meet this definition were more likely to have “alcohol consumption which raises the risk of lifestyle diseases,” as shown in Health Japan 21 (the second term), a national health promotion initiative.^8^^,^^9^ A number of variables in the JMDC-SHC database were not compared with the NHNS data because they were defined differently or were not covered by the NHNS; namely, regular exercise, dietary habits, drinking habits (alcohol drinking frequency and excessive drinking), and sleep habits.

We defined lifestyle diseases (MS, hypertension, dyslipidemia, and diabetes) according to the criteria of the NHNS.^5^ MS was defined both by the presence of an excessive WC (≥85 cm in men and ≥90 cm in women) and by meeting two or more of the following criteria: (1) SBP >130 mm Hg and/or DBP >85 mm Hg and/or the use of antihypertensive drugs; (2) HDL-C <40 mg/dL and/or the use of antihyperlipidemic drugs; and (3) HbA1c ≥6.0% and/or the use of antidiabetic drugs. Hypertension was defined as SBP ≥140 mm Hg and/or DBP ≥90 mm Hg and/or the use of antihypertensive drugs. Dyslipidemia was defined as HDL-C <40 mg/dL and/or the use of antihyperlipidemic drugs. Diabetes was defined as HbA1c ≥6.5% and/or the use of antidiabetic drugs. Drug treatment for any of the above three diseases (hypertension, dyslipidemia, and diabetes) was defined as the use of drugs for these conditions.

### Statistical analysis

The subject characteristics, prevalence of lifestyle behaviors, lifestyle diseases, drug treatment for lifestyle diseases, and number of MS components were summarized for each sex-age group (40–49, 50–59, 60–69, and 70–74 years). The prevalence of drug treatment for lifestyle diseases was determined as the percentage of people who took drugs for these conditions among those who met the respective lifestyle disease definition. We used the results of the lifestyle survey and physical examination of NHNS subjects aged ≥40 years as the national benchmark for comparison. Data are presented as the mean and standard deviation (SD) for continuous variables and percentages for categorical variables. All statistical analyses were performed with SAS version 9.4 (SAS Institute Inc., Cary, NC, USA).

## RESULTS

### Characteristics of study subjects

The JMDC-SHC database was composed of a total of 646,869 subjects, of whom 66.5% were men with a mean age of 50.9 (SD, 7.6) years and 33.5% were women with a mean age of 50.6 (SD, 7.7) years, with an age range of 40–74 years. Of the total, 99.8% of men and 41.5% of women were employees (Table 1). The largest age group was 40–49 years in both men (48.3%) and women (51.1%), while those aged 70 years or older accounted for 1.3% of men and 1.6% of women. As age increased, SBP (men, 122.4 mm Hg in age 40s to 130.2 mm Hg in age 70s; women, 113.6 mm Hg in age 40s to 131.0 mm Hg in age 70s) and HbA1c (men, 5.5% in age 40s to 5.9% in age 70s; women, 5.4% in age 40s to 5.9% in age 70s) in the SHC subjects increased in both sexes, as did WC in women (Table 2). Overweight (BMI ≥25 kg/m^2^) in men decreased in the older age groups (32.5% in age 40s, 23.4% in age 70s). Underweight (BMI <18.5 kg/m^2^) was most prevalent in women in their 40s (14.5%) and gradually decreased with aging.

**Table 1.** Sex and age distributions of the Specific Health Checkup group

Age group, years	Total		Employees		Dependents	
Men						
40–49	207,532	(48.3)	207,416	(48.3)	116	(13.4)
50–59	158,017	(36.7)	157,884	(36.8)	133	(15.3)
60–69	59,005	(13.7)	58,575	(13.7)	430	(49.5)
70–74	5,549	(1.3)	5,360	(1.3)	189	(21.8)
Total	430,103	(100)	429,235	(100)	868	(100)
Women						
40–49	110,806	(51.1)	50,279	(55.8)	60,527	(47.8)
50–59	74,303	(34.3)	29,741	(33.0)	44,562	(35.2)
60–69	28,181	(13.0)	9,686	(10.8)	18,495	(14.6)
70–74	3,476	(1.6)	336	(0.4)	3,140	(2.5)
Total	216,766	(100)	90,042	(100)	126,724	(100)

Data are presented as number of subjects (%).

**Table 2.** Characteristics according to age and sex in the Specific Health Checkup group and the National Health and Nutrition Survey group^a^

	Specific Health Checkup group						National Health and Nutrition Survey group					
	
	Total	Age group, years				Missing values	Total	Age group, years				Missing values
	
		40–49	50–59	60–69	70–74			40–49	50–59	60–69	≥70	
Men												
Number of subjects	430,103	207,532	158,017	59,005	5,549		2,032	382	378	589	683	
BMI, kg/m^2^	23.9 (3.5)	23.9 (3.7)	23.9 (3.3)	23.5 (3.0)	23.1 (2.7)	1,415 (0.3)	N.A.	24.2 (3.8)	23.8 (3.3)	23.7 (3.1)	23.2 (3.0)	18 (0.9)
BMI < 18.5	12,748 (3.0)	6,262 (3.0)	4,349 (2.8)	1,934 (3.3)	203 (3.7)		77 (3.8)	10 (2.7)	10 (2.7)	21 (3.6)	36 (5.3)	
18.5 ≤ BMI < 25	279,166 (65.1)	133,356 (64.4)	102,067 (64.8)	39,731 (67.8)	4,012 (72.9)		1,341 (66.6)	228 (60.8)	239 (64.1)	391 (66.8)	483 (70.9)	
25 ≤ BMI	136,774 (31.9)	67,319 (32.5)	51,213 (32.5)	16,953 (28.9)	1,289 (23.4)		596 (29.6)	137 (36.5)	124 (33.2)	173 (29.6)	162 (23.8)	
WC, cm	84.5 (9.2)	84.1 (9.6)	85.0 (8.9)	84.8 (8.3)	83.8 (7.7)	1,626 (0.4)	N.A.	N.A.	N.A.	N.A.	N.A.	N.A.
SBP, mm Hg	124.8 (15.6)	122.4 (14.6)	126.0 (15.8)	129.8 (17.0)	130.2 (17.5)	1,426 (0.3)	N.A.	125.7 (14.8)	133.1 (17.3)	137.9 (17.1)	137.8 (16.5)	780 (38.4)
DBP, mm Hg	78.5 (11.1)	77.0 (11.2)	80.0 (11.0)	79.6 (10.7)	76.3 (10.7)	1,426 (0.3)	N.A.	84.3 (10.8)	85.1 (10.8)	84.0 (11.1)	78.5 (10.7)	780 (38.4)
HDL-C, mg/dL	58.5 (15.4)	57.7 (14.9)	59.2 (15.8)	59.6 (15.9)	59.6 (15.8)	1,693 (0.4)	N.A.	57.0 (14.2)	56.2 (14.6)	57.0 (16.2)	52.7 (14.9)	867 (42.7)
HbA1c, %	5.6 (0.7)	5.5 (0.6)	5.7 (0.7)	5.8 (0.7)	5.9 (0.7)	69,760 (16.2)	N.A.	5.5 (0.4)	5.9 (1.0)	6.0 (0.8)	6.0 (0.7)	875 (43.1)
Women												
Number of subjects	216,766	110,806	74,303	28,181	3,476		2,536	536	478	705	817	
BMI, kg/m^2^	22.0 (3.7)	21.8 (3.8)	22.0 (3.7)	22.3 (3.5)	22.5 (3.4)	6,327 (2.9)	N.A.	22.3 (3.9)	22.4 (4.0)	22.7 (3.4)	22.8 (3.6)	18 (0.7)
BMI < 18.5	28,402 (13.5)	15,589 (14.5)	9,502 (13.1)	2,989 (10.9)	322 (9.9)		242 (9.6)	53 (10.0)	55 (11.6)	47 (6.7)	87 (10.7)	
18.5 ≤ BMI < 25	145,802 (69.3)	74,327 (69.3)	50,072 (69.0)	19,118 (69.8)	2,285 (70.1)		1733 (68.8)	378 (71.2)	323 (67.9)	501 (71.6)	531 (65.5)	
25 ≤ BMI	36,235 (17.2)	17,319 (16.2)	12,981 (17.9)	5,281 (19.3)	654 (20.1)		543 (21.6)	100 (18.8)	98 (20.6)	152 (21.7)	193 (23.8)	
WC, cm	78.3 (9.6)	77.2 (9.5)	79.0 (9.7)	80.8 (9.4)	82.0 (9.4)	6,646 (3.1)	N.A.	N.A.	N.A.	N.A.	N.A.	N.A.
SBP, mm Hg	117.7 (17.4)	113.6 (15.6)	119.9 (17.7)	126.2 (18.2)	131.0 (18.0)	6,387 (2.9)	N.A.	118.1 (15.7)	127.2 (18.5)	132.5 (16.7)	137.8 (17.9)	739 (29.1)
DBP, mm Hg	71.6 (11.5)	69.6 (11.2)	73.3 (11.7)	74.7 (11.1)	74.2 (10.9)	6,387 (2.9)	N.A.	75.4 (10.5)	79.8 (10.9)	79.7 (10.3)	76.6 (10.4)	739 (29.1)
HDL-C, mg/dL	71.9 (16.7)	71.0 (15.9)	73.8 (17.4)	70.7 (17.1)	67.8 (16.4)	6,387 (2.9)	N.A.	68.3 (15.7)	69.8 (17.3)	65.2 (16.7)	60.8 (15.3)	848 (33.4)
HbA1c, %	5.5 (0.5)	5.4 (0.4)	5.6 (0.5)	5.8 (0.6)	5.9 (0.6)	32,037 (14.8)	N.A.	5.6 (0.4)	5.8 (0.7)	5.9 (0.6)	6.0 (0.7)	853 (33.6)

BMI, body mass index; WC, waist circumference; SBP, systolic blood pressure; DBP, diastolic blood pressure; HDL-C, high-density lipoprotein cholesterol; HbA1c, hemoglobin A1c.

Data are presented as mean (SD) or number of subjects (%).

^a^Data were obtained from official reports of the 2015 National Health and Nutrition Survey.^5^

### Lifestyle behaviors

Table 3 and Table 4 show the prevalence of lifestyle behaviors by age group in men and women. Current smoking was 41.0% in men and 10.2% in women in their 40s and decreased as age advanced (16.2% in men and 4.4% in women in their 70s). Eating habits, regular exercise, and sleep habits became more favorable as age advanced. In men, excessive drinking increased with advancing age, reaching 60.6% among men in their 50s, then gradually decreasing at age 60–74 years. In women, excessive drinking decreased from 27.0% in their 40s to 11.4% in their 70s.

**Table 3.** Prevalence of lifestyle behaviors according to age in men in the Specific Health Checkup group and National Health and Nutrition Survey group^a^

	Specific Health Checkup group						National Health and Nutrition Survey group					
	
	Total	Age group, years				Missing values	Total	Age group, years				Missing values
	
		40–49	50–59	60–69	70–74			40–49	50–59	60–69	≥70	
Number of subjects	430,103	207,532	158,017	59,005	5,549		2,599	553	519	713	814	
Current smoking	163,126 (38.2)	84,565 (41.0)	59,624 (38.0)	18,044 (30.9)	893 (16.2)	3,026 (0.7)	733 (28.3)	208 (37.7)	193 (37.2)	209 (29.4)	123 (15.2)	6 (0.2)
Regular exercise	88,119 (22.9)	35,722 (19.2)	32,131 (22.8)	17,248 (32.7)	3,018 (58.5)	44,994 (10.5)						
Dietary habits												
Eating speed						49,381 (11.5)						N.A.
Slow	32,639 (8.6)	14,742 (8.0)	12,361 (8.9)	5,029 (9.6)	507 (9.9)		N.A.	N.A.	N.A.	N.A.	N.A.	
Normal	221,224 (58.1)	102,815 (56.0)	82,246 (59.0)	32,756 (62.5)	3,407 (66.2)		N.A.	N.A.	N.A.	N.A.	N.A.	
Fast	126,859 (33.3)	66,187 (36.0)	44,826 (32.2)	14,615 (27.9)	1231 (23.9)		N.A.	N.A.	N.A.	N.A.	N.A.	
Frequent skipping breakfast	72,361 (18.8)	44,280 (23.9)	23,039 (16.4)	4,846 (9.2)	196 (3.8)	46,107 (10.7)	N.A.	N.A.	N.A.	N.A.	N.A.	N.A.
Eating dinner late	151,780 (40.6)	85,702 (47.6)	53,633 (39.3)	11,816 (22.8)	629 (12.2)	56,507 (13.1)	N.A.	N.A.	N.A.	N.A.	N.A.	N.A.
Frequent snacking	56,362 (14.7)	30,843 (16.6)	19,346 (13.7)	5,816 (11.0)	357 (6.9)	45,339 (10.5)	N.A.	N.A.	N.A.	N.A.	N.A.	N.A.
Drinking habits												
Alcohol drinking frequency						26,440 (6.1)						N.A.
Rare	125,038 (31.0)	65,178 (33.4)	42,251 (28.7)	15,899 (28.5)	1,710 (31.9)		N.A.	N.A.	N.A.	N.A.	N.A.	
Occasional	132,721 (32.9)	69,697 (35.7)	46,880 (31.8)	14,876 (26.6)	1,268 (23.7)		N.A.	N.A.	N.A.	N.A.	N.A.	
Everyday	145,904 (36.2)	60,237 (30.9)	58,231 (39.5)	25,061 (44.9)	2,375 (44.4)		N.A.	N.A.	N.A.	N.A.	N.A.	
Alcohol consumption						119,233 (27.7)						948 (36.5)
<1 glass of sake	127,872 (41.1)	64,706 (43.8)	43,056 (37.8)	17,891 (39.7)	2,219 (51.3)		500 (30.3)	76 (22.7)	69 (18.6)	132 (27.0)	223 (48.8)	
1–2 glass of sake	110,268 (35.5)	50,234 (34.0)	41,518 (36.4)	16,982 (37.7)	1,534 (35.4)		651 (39.4)	125 (37.3)	160 (43.1)	197 (40.4)	169 (37.0)	
2–3 glass of sake	55,254 (17.8)	23,710 (16.1)	22,561 (19.8)	8,472 (18.8)	511 (11.8)		338 (20.5)	70 (20.9)	100 (27.0)	120 (24.6)	48 (10.5)	
≥3 glass of sake	17,476 (5.6)	8,934 (6.1)	6,795 (6.0)	1,681 (3.7)	66 (1.5)		162 (9.8)	64 (19.1)	42 (11.3)	39 (8.0)	17 (3.7)	
Excessive drinking	175,797 (57.0)	78,876 (53.9)	68,426 (60.6)	26,430 (59.0)	2,065 (47.8)	121,647 (28.3)	N.A.	N.A.	N.A.	N.A.	N.A.	N.A.
Sleep habits	211,406 (56.9)	95,360 (53.3)	76,034 (56.1)	35,833 (69.4)	4,179 (81.2)	58,724 (13.7)	N.A.	N.A.	N.A.	N.A.	N.A.	N.A.

Data are presented as number of subjects (%).

^a^Data were obtained from official reports of the 2015 National Health and Nutrition Survey.^5^

**Table 4.** Prevalence of lifestyle behaviors according to age in women in the Specific Health Checkup group and National Health and Nutrition Survey group^a^

	Specific Health Checkup group						National Health and Nutrition Survey group					
	
	Total	Age group, years				Missing values	Total	Age group, years				Missing values
	
		40–49	50–59	60–69	70–74			40–49	50–59	60–69	≥70	
Number of subjects	216,766	110,806	74,303	28,181	3,476		3,081	659	586	830	1,006	
Current smoking	20,212 (9.5)	11,149 (10.2)	6,845 (9.4)	2,070 (7.5)	148 (4.4)	3,759 (1.7)	234 (7.6)	77 (11.7)	65 (11.1)	69 (8.3)	23 (2.3)	5 (0.2)
Regular exercise	40,068 (20.3)	14,551 (14.3)	15,476 (22.8)	8,522 (34.1)	1,519 (49.0)	19,148 (8.8)						
Dietary habits												
Eating speed						21,245 (9.8)						N.A.
Slow	17,684 (9.0)	9,913 (9.9)	5,619 (8.4)	1,889 (7.5)	263 (8.4)		N.A.	N.A.	N.A.	N.A.	N.A.	
Normal	128,118 (65.5)	64,307 (64.3)	44,464 (66.2)	17,132 (67.8)	2,215 (71.0)		N.A.	N.A.	N.A.	N.A.	N.A.	
Fast	49,719 (25.4)	25,762 (25.8)	17,072 (25.4)	6,245 (24.7)	640 (20.5)		N.A.	N.A.	N.A.	N.A.	N.A.	
Frequent skipping breakfast	19,573 (9.9)	11,971 (11.8)	6,024 (8.9)	1,466 (5.9)	112 (3.6)	19,431 (9.0)	N.A.	N.A.	N.A.	N.A.	N.A.	N.A.
Eating dinner late	32,240 (16.3)	18,534 (18.4)	10,645 (15.7)	2,798 (11.0)	263 (8.3)	19,386 (8.9)	N.A.	N.A.	N.A.	N.A.	N.A.	N.A.
Frequent snacking	36,632 (18.4)	19,598 (19.2)	12,943 (18.9)	3,875 (15.2)	216 (6.8)	17,659 (8.1)	N.A.	N.A.	N.A.	N.A.	N.A.	N.A.
Drinking habits												
Alcohol drinking frequency						13,676 (6.3)						N.A.
Rare	121,598 (59.9)	60,027 (57.8)	41,620 (59.7)	17,468 (66.3)	2,483 (76.6)		N.A.	N.A.	N.A.	N.A.	N.A.	
Occasional	57,453 (28.3)	31,500 (30.3)	19,611 (28.1)	5,817 (22.1)	525 (16.2)		N.A.	N.A.	N.A.	N.A.	N.A.	
Everyday	24,039 (11.8)	12,289 (11.8)	8,454 (12.1)	3,064 (11.6)	232 (7.2)		N.A.	N.A.	N.A.	N.A.	N.A.	
Alcohol consumption						89,499 (41.3)						2,162 (70.2)
<1 glass of sake	93,017 (73.1)	47,518 (71.1)	32,148 (73.5)	12,027 (79.3)	1,324 (87.5)		539 (58.7)	114 (42.5)	124 (52.1)	152 (63.9)	149 (85.1)	
1–2 glass of sake	25,325 (19.9)	13,861 (20.7)	8,790 (20.1)	2,515 (16.6)	159 (10.5)		269 (29.3)	105 (39.2)	79 (33.2)	69 (29.0)	16 (9.1)	
2–3 glass of sake	7,123 (5.6)	4,294 (6.4)	2,275 (5.2)	528 (3.5)	26 (1.7)		72 (7.8)	30 (11.2)	23 (9.7)	12 (5.0)	7 (4.0)	
≥3 glass of sake	1,802 (1.4)	1,176 (1.8)	518 (1.2)	104 (0.7)	4 (0.3)		39 (4.2)	19 (7.1)	12 (5.0)	5 (2.1)	3 (1.7)	
Excessive drinking	31,904 (25.2)	17,905 (27.0)	10,869 (25.0)	2,959 (19.6)	171 (11.4)	90,299 (41.7)	N.A.	N.A.	N.A.	N.A.	N.A.	N.A.
Sleep habits	108,594 (56.3)	54,071 (54.3)	36,099 (55.0)	16,133 (65.9)	2,291 (72.9)	23,903 (11.0)	N.A.	N.A.	N.A.	N.A.	N.A.	N.A.

Data are presented as number of subjects (%).

^a^Data were obtained from official reports of the 2015 National Health and Nutrition Survey.^5^

### Metabolic syndrome components

Table 5 shows the distribution of MS components by age group, sex, and WC. In SHC subjects with more than one MS component, the number of people with an excessive WC was greater than that with a normal WC in both sexes across all age groups, especially among those aged 40–49 years (20.1% vs 3.7% in men, 16.5% vs 1.3% in women). The mean number of MS components increased with age regardless of sex or WC.

**Table 5.** Distribution of the number of metabolic syndrome components according to age, sex and waist circumference in the Specific Health Checkup group and National Health and Nutrition Survey group^a^

Specific Health Checkup group								National Health and Nutrition Survey group							
	
Age group, years	WC	*N*	MS components (mean)	Number of MS components				Age group, years	WC	*N*	MS components (mean)	Number of MS components			
	
				0	1	2	3					0	1	2	3
Men															
Total	<85 cm	184,878	0.6	101,256 (54.8)	66,384 (35.9)	14,624 (7.9)	2,614 (1.4)	Total	<85 cm	461	1.1	112 (24.3)	213 (46.2)	104 (22.6)	32 (6.9)
	≥85 cm	148,140	1.1	42,373 (28.6)	62,687 (42.3)	32,107 (21.7)	10,973 (7.4)		≥85 cm	691	1.6	62 (9.0)	259 (37.5)	234 (33.9)	136 (19.7)
40–49	<85 cm	93,818	0.4	63,569 (67.8)	26,733 (28.5)	3,210 (3.4)	306 (0.3)	40–49	<85 cm	69	0.4	40 (58.0)	28 (40.6)	1 (1.4)	0 (0.0)
	≥85 cm	67,720	0.9	25,779 (38.1)	28,360 (41.9)	10,766 (15.9)	2,815 (4.2)		≥85 cm	80	1.0	22 (27.5)	42 (52.5)	10 (12.5)	6 (7.5)
50–59	<85 cm	64,556	0.7	30,027 (46.5)	27,059 (41.9)	6,370 (9.9)	1,100 (1.7)	50–59	<85 cm	62	0.9	18 (29.0)	35 (56.5)	7 (11.3)	2 (3.2)
	≥85 cm	56,626	1.2	13,214 (23.3)	24,380 (43.1)	13,941 (24.6)	5,091 (9.0)		≥85 cm	102	1.4	17 (16.7)	40 (39.2)	34 (33.3)	11 (10.8)
60–69	<85 cm	24,292	1.0	7,202 (29.6)	11,545 (47.5)	4,471 (18.4)	1,074 (4.4)	60–69	<85 cm	139	1.2	28 (20.1)	63 (45.3)	42 (30.2)	6 (4.3)
	≥85 cm	22,046	1.4	3,219 (14.6)	9,294 (42.2)	6,759 (30.7)	2,774 (12.6)		≥85 cm	240	1.7	12 (5.0)	94 (39.2)	87 (36.3)	47 (19.6)
70–74	<85 cm	2,212	1.2	458 (20.7)	1,047 (47.3)	573 (25.9)	134 (6.1)	≥70	<85 cm	191	1.4	26 (13.6)	87 (45.5)	54 (28.3)	24 (12.6)
	≥85 cm	1,748	1.6	161 (9.2)	653 (37.4)	641 (36.7)	293 (16.8)		≥85 cm	269	1.9	11 (4.1)	83 (30.9)	103 (38.3)	72 (26.8)
Women															
Total	<90 cm	150,681	0.4	104,012 (69.0)	36,912 (24.5)	7,996 (5.3)	1,761 (1.2)	Total	<90 cm	1,269	1.0	453 (35.7)	499 (39.3)	236 (18.6)	81 (6.4)
	≥90 cm	19,376	1.0	6,157 (31.8)	7,670 (39.6)	4,096 (21.1)	1,453 (7.5)		≥90 cm	403	1.6	50 (12.4)	147 (36.5)	129 (32.0)	77 (19.1)
40–49	<90 cm	78,473	0.2	64,961 (82.8)	12,442 (15.9)	967 (1.2)	103 (0.1)	40–49	<90 cm	274	0.4	195 (71.2)	63 (23.0)	15 (5.5)	1 (0.4)
	≥90 cm	8,338	0.8	3,713 (44.5)	3,247 (38.9)	1,126 (13.5)	252 (3.0)		≥90 cm	52	0.9	17 (32.7)	23 (44.2)	11 (21.2)	1 (1.9)
50–59	<90 cm	51,843	0.5	31,713 (61.2)	16,180 (31.2)	3,357 (6.5)	593 (1.1)	50–59	<90 cm	241	0.7	107 (44.4)	110 (45.6)	22 (9.1)	2 (0.8)
	≥90 cm	7,196	1.1	1,896 (26.3)	3,018 (41.9)	1,695 (23.6)	587 (8.2)		≥90 cm	65	1.4	10 (15.4)	25 (38.5)	25 (38.5)	5 (7.7)
60–69	<90 cm	18,432	0.9	6,926 (37.6)	7,520 (40.8)	3,129 (17.0)	857 (4.6)	60–69	<90 cm	391	1.1	110 (28.1)	164 (41.9)	87 (22.3)	30 (7.7)
	≥90 cm	3,378	1.5	502 (14.9)	1,264 (37.4)	1,083 (32.1)	529 (15.7)		≥90 cm	117	1.6	13 (11.1)	44 (37.6)	32 (27.4)	28 (23.9)
70–74	<90 cm	1,933	1.3	412 (21.3)	770 (39.8)	543 (28.1)	208 (10.8)	≥70	<90 cm	363	1.5	41 (11.3)	162 (44.6)	112 (30.9)	48 (13.2)
	≥90 cm	464	1.7	46 (9.9)	141 (30.4)	192 (41.4)	85 (18.3)		≥90 cm	169	1.8	10 (5.9)	55 (32.5)	61 (36.1)	43 (25.4)

WC, waist circumference; MS, metabolic syndrome.

Data are presented as number of subjects (%).

^a^Data were obtained from official reports of the 2015 National Health and Nutrition Survey.^5^

### Lifestyle diseases and drug treatment

The prevalence of lifestyle diseases and therapeutic treatment is shown in Table 6. Overall, trends in the prevalence of lifestyle diseases increased with advancing age. The prevalence of MS increased steadily as age advanced, reaching 23.6% and 11.6% in men and women aged 70–74 years, respectively. The prevalence of hypertension exceeded 50% in men aged 60–74 years and women aged 70–74 years. The prevalence of dyslipidemia increased with age and reached 28.5% and 37.1% in men and women aged 70–74 years, respectively. The prevalence of diabetes was 4.8% and 1.5% in men and women aged 40–49 years, respectively, and steadily increased to 19.4% and 12.9% in men and women aged 70–74 years, respectively.

**Table 6.** Prevalence of lifestyle diseases and drug treatment for them according to age and sex in the Specific Health Checkup group and National Health and Nutrition Survey group^a^

	Specific Health Checkup group						National Health and Nutrition Survey group					
	
	Total	Age group, years				Missing values	Total	Age group, years				Missing values
	
		40–49	50–59	60–69	70–74			40–49	50–59	60–69	≥70	
Men												
No. of subjects	430,103	207,532	158,017	59,005	5,549		2032	382	378	589	683	
Metabolic syndrome	43,080 (12.9)	13,581 (8.4)	19,032 (15.7)	9,533 (20.6)	934 (23.6)	97,085 (22.6)	370 (32.1)	16 (10.7)	45 (27.4)	134 (35.4)	175 (38.0)	880 (43.3)
Hypertension	125,151 (31.2)	38,165 (19.9)	55,325 (37.3)	28,573 (51.5)	3,088 (57.0)	28,835 (6.7)	789 (63.1)	59 (35.8)	94 (52.8)	270 (67.3)	366 (72.2)	781 (38.4)
Drug treatment for hypertension	66,796 (53.4)	13,921 (36.5)	31,062 (56.1)	19,471 (68.1)	2,342 (75.8)		473 (59.9)	17 (28.8)	49 (52.1)	141 (52.2)	266 (72.7)	
Dyslipidemia	65,225 (16.3)	23,467 (12.2)	27,336 (18.4)	12,877 (23.2)	1,545 (28.5)	29,117 (6.8)	326 (28.1)	14 (9.2)	35 (21.3)	111 (29.1)	166 (35.9)	870 (42.8)
Drug treatment for dyslipidemia	40,227 (61.7)	9,912 (42.2)	18,953 (69.3)	10,092 (78.4)	1,270 (82.2)		209 (64.1)	9 (64.3)	25 (71.4)	75 (67.6)	100 (60.2)	
Diabetes	28,914 (8.7)	7,835 (4.8)	12,900 (10.6)	7,412 (16.0)	767 (19.4)	96,978 (22.5)	255 (22.0)	11 (7.3)	31 (18.8)	87 (22.9)	126 (27.3)	875 (43.1)
Drug treatment for diabetes	18,256 (63.1)	4,440 (56.7)	8,327 (64.6)	5,005 (67.5)	484 (63.1)		143 (56.1)	4 (36.4)	13 (41.9)	49 (56.3)	77 (61.1)	
Women												
No. of subjects	216,766	110,806	74,303	28,181	3,476		2,536	536	478	705	817	
Metabolic syndrome	5,549 (3.3)	1,378 (1.6)	2,282 (3.9)	1,612 (7.4)	277 (11.6)	46,709 (21.5)	206 (12.3)	12 (3.7)	30 (9.8)	60 (11.8)	104 (19.5)	864 (34.1)
Hypertension	34,743 (17.8)	8,763 (8.8)	14,722 (21.7)	9,608 (37.5)	1,650 (53.7)	21,266 (9.8)	835 (46.5)	54 (15.7)	109 (33.1)	260 (48.1)	412 (70.9)	740 (29.2)
Drug treatment for hypertension	17,738 (51.1)	2,880 (32.9)	7,571 (51.4)	6,074 (63.2)	1,213 (73.5)		512 (61.3)	14 (25.9)	43 (39.4)	141 (54.2)	314 (76.2)	
Dyslipidemia	17,244 (8.8)	2,704 (2.7)	7,066 (10.4)	6,332 (24.7)	1,142 (37.1)	21,285 (9.8)	397 (23.6)	20 (6.1)	34 (11.1)	141 (27.6)	202 (37.4)	851 (33.6)
Drug treatment for dyslipidemia	15,560 (90.2)	1,754 (64.9)	6,598 (93.4)	6,097 (96.3)	1,111 (97.3)		354 (89.2)	13 (65.0)	28 (82.4)	132 (93.6)	181 (89.6)	
Diabetes	5,682 (3.3)	1,283 (1.5)	2,272 (3.8)	1,817 (8.3)	310 (12.9)	46,411 (21.4)	179 (10.6)	9 (2.8)	20 (6.5)	58 (11.4)	92 (17.2)	855 (33.7)
Drug treatment for diabetes	3,302 (58.1)	727 (56.7)	1,298 (57.1)	1,084 (59.7)	193 (62.3)		94 (52.5)	4 (44.4)	6 (30.0)	28 (48.3)	56 (60.9)	

Data are presented as number of subjects (%).

The prevalence of drug treatment for lifestyle diseases was calculated as the percentage of people who took drugs for them among those met the lifestyle disease definitions.

^a^Data were obtained from official reports of the 2015 National Health and Nutrition Survey.^5^

In the SHC group, the prevalence of drug treatment for hypertension and dyslipidemia increased with advancing age in both sexes. The prevalence of drug treatment for diabetes was about 60% regardless of age or sex group.

### Comparison with the results of the National Health and Nutrition Survey

The most prevalent age group in the SHC was 40–49 years, whereas that in the NHNS was ≥70 years (Table 1 and eTable 2). In the SHC group, a mean SBP of more than 130 mm Hg was observed in men and women aged 70–74 years, while in the NHNS group, this was observed in men aged ≥50 years and women aged ≥60 years (Table 2). The proportion of underweight (BMI <18.5 kg/m^2^) in women aged 40–60 years was higher in the SHC group than in the NHNS group.

Compared with the NHNS group, the prevalence of smoking in the SHC group was slightly higher in men across all age groups. A lower prevalence of smoking in women was observed in both the SHC and NHNS groups (Table 3 and Table 4). Overall, alcohol intake was higher in the NHNS group than the SHC group.

Although the mean number of MS components was lower in the SHC group than in the NHNS group, we found a similar increasing trend in the number of MS components with age in both groups (Table 5). Overall, the numbers of male subjects with no MS components were larger in the SHC groups than in the NHNS groups. The difference was most prominent among those with a WC <85 cm in men or <90 cm in women in their 50’s (men, 46.5% in the SHC group vs 29.0% in the NHNS group; women, 61.2% in the SHC group vs 44.4% in the NHNS group).

The SHC group had a lower prevalence of MS than the NHNS group in both sexes; in particular, the difference was 12–15% in men aged >50 years (Table 6). Regardless of age, the NHNS groups had a higher prevalence of hypertension, by more than 15% in men. The SHC women had a lower prevalence of hypertension for the respective NHNS groups, and the difference increased with advancing age. The SHC men in their 40s had a 3% higher prevalence of dyslipidemia than their peer NHNS men, but a lower prevalence in the other age deciles of the SHC group. The SHC women had lower prevalence of dyslipidemia up to 3% than their peer NHNS women. More than half of diabetes patients had drug treatment in the NHNS group.

The number of SHC hypertension patients treated with drugs was larger than that of NHNS hypertension patients across all age groups, except women aged >70 years (76.2% in the NHNS group vs 73.5% in the SHC group) (Table 6). The portion of male SHC patients treated for dyslipidemia increased with increasing age, whereas the proportion among NHNS patients stayed around 60.2–71.4%, with no increasing trend. In women, treated dyslipidemia patients reached about 65% in both the 40–49 years SHC and NHNS groups, then rose to 93–97% and 82–94% at >50 years in the SHC and NHNS groups, respectively.

## DISCUSSION

In the present study, we investigated lifestyle behaviors and lifestyle diseases among insured people using the JMDC-SHC database and assessed the utility of the database in highlighting the actual health condition of company employees and their dependents. Findings were contrasted with the NHNS results. Results showed significant differences between them, indicating that use of the NHNS as a data source may not be suitable for the health management of employees and their dependents. The possibility of using the JMDC-SHC database for this purpose should be further explored.

The differences between these two entities may primarily derive from the difference in their sampled populations. The NHNS group was composed of a general population aged over 20 years via stratified random sampling from every prefecture in Japan. However, the sample size of the NHNS was much smaller than that of the JMDC-SHC database. Further, the NHNS data may have suffered from significant selection bias due to the low response rate to the NHNS (dietary intake survey: 59–67%, lifestyle survey: 59–68%, physical examination: 49–57%, blood test: 32–39%).^10^^,^^11^

In the present study, increasing trends in the prevalence of lifestyle diseases were observed as age advanced, as has also been reported in other studies on MS,^12^^,^^13^ hypertension,^13^^,^^14^ dyslipidemia,^13^^,^^14^ and diabetes.^13^^–^^15^ On comparison among the same age groups, the mean number of MS components and prevalence of all lifestyle diseases were generally lower in the SHC group than in the NHNS group. The lower mean BP in the SHC group than in the NHNS group is consistent with the findings of a recent retrospective, cross-sectional study among Japanese employees using a nationwide healthcare database in Japan,^16^ and may be explained as follows: first, the healthy worker effect is likely to be present in the SHC group.^17^ The JMDC-SHC database consists only of employees and their dependents covered by the employee’s health insurance and is, therefore, dominated by a working-age population. Because employees have to be healthy to maintain their involvement in the workforce, the working population is known to be healthier than the general population. In contrast, the NHNS group was randomly selected from the general population, and included subscribers of other types of health insurance plans and non-workers. Second, company health insurance societies, whose beneficiaries are the JMDC database enrollees, appear to offer better healthcare services beyond the regular, mandatory services provided by other types of health insurers, owing to their typically better funding.

The prevalence of drug treatment for hypertension and dyslipidemia was generally higher in the SHC group than in the NHNS group. The likely reason for this is that supervisors and human resource departments at workplaces are obliged to monitor whether their ill employees are undergoing treatment appropriately, in collaboration with health insurance societies. We did not compare the prevalence of drug treatment for diabetes between the SHC and NHNS groups because of the very few patients with diabetes in the NHNS group.

The present results indicate that subjects aged 40–49 years in both the SHC and NHNS groups were most likely to have unhealthy lifestyle behaviors, potentially increasing their subsequent risk of lifestyle diseases.^18^^–^^22^ It is, therefore, imperative to intervene with this at-risk population to prevent the further development of lifestyle diseases in their later years. The SHC group had generally adopted healthier behaviors than the NHNS group, probably due to the healthy worker effect. However, regarding underweight in women, this was more prevalent in the SHC group than NHNS group in those aged 40–59 years. A previous pooled analysis reported that underweight in Japanese was associated with risk of mortality.^23^ The present Specific Health Guidance is targeted for people with BMI ≥25 kg/m^2^ but should be extended for those with BMI <18.5 kg/m^2^ to provide them with nutritional guidance.

Our study has three limitations. First, the prevalence of lifestyle behaviors and lifestyle diseases might have been underestimated because missing observations were not included in the analysis. Second, we relied on interview records to determine the presence of treatment with therapeutic drugs for lifestyle diseases. Some subjects may not have answered honestly or may not have correctly understood what kind of treatment they were receiving; if so, prevalence in the present study may have been underestimated. Third, we could not examine differences in occupation, socioeconomic status, and region between the SHC and NHNS groups because this information was not available. Accordingly, we were also unable to assess the external validity of the study by applying the present findings to the population of all health insurance societies. In addition, the findings of this study might not be applicable to subscribers of other health insurance providers, such as the Japan Health Insurance Association, Mutual Aid Association, and National Health Insurance.

Further, the following points warrant noting as limitations of the JMDC-SHC database. First, the healthy worker effect might be prominent when compared with the entire working population. This effect can result in biased estimation of the prevalence and incidence of lifestyle diseases. For example, a previous review reported that health-conscious people who receive preventive therapy (eg, statin therapy) are more likely to adopt healthier behaviors, so they may have a lower incidence of a related outcome (eg, myocardial infarction).^24^ The enrollees of the JMDC-SHC database were employees of large companies and their dependents, and are, therefore, likely to be highly educated and health-conscious and have sufficient income to access quality health services. Second, individuals in the JMDC-SHC database cannot be tracked if they change their insurance plan due to a change in employment, or following resignation, retirement, or reaching the age of 75 and entering the government-run medical care system for elderly in the latter stage of life. Thus, the limited availability of outcomes requiring long-term follow-up, which mostly occur in later life stages, such as ischemic heart disease and stroke, should be noted.

In summary, we clarified the difference between findings obtained using the JMDC-SHC database and NHNS results. For the healthcare management of employees from the viewpoint of employers and payers, which are required to implement the Data Health Plan by the Government, we consider that the JMDC-SHC database will serve as an important reference benchmark rather than the NHNS. This is because the former consists of a subpopulation of working generations covered by company insurance plans, whereas the latter has a different age distribution from the working population. On this basis, current workforce healthcare strategies based on the data of the NHNS should be reconsidered. The JMDC-SHC database should be further studied for its potential as a valid reference for use in the development of effective healthcare management strategies for the prevention of lifestyle diseases.

## APPENDIX A. SUPPLEMENTARY DATA

eTable 1. Self-administered questionnaire on lifestyle for Specific Health Checkups

eTable 2. Sex and age distributions of respondents to the National Health and Nutrition Survey group^a^
