# Supplementary material for: Utility of a Specific Health Checkup Database Containing Lifestyle Behaviors and Lifestyle Diseases for Employee Health Insurance in Japan
Source: J Epidemiol. 2020 Feb 5;30(2):57–66. doi: 10.2188/jea.JE20180192 (PMC6949185; doi:10.2188/jea.JE20180192)
Supplement: Supplementary file 1 [file je-30-057-s001.pdf]

eTable 1. Self-administered questionnaire on lifestyle for Specific Health Checkups

Lifestyle behaviors	Questionnaire items	Answer choice
Current smoking	Are you a current regular smoker? (A "current regular smoker" refers to those who has smoked 100 or more cigarettes or smoked for at least six months and has been smoking over the past month.)	Yes or No
Regular exercise	Have you been in the habit of doing exercise for thirty minutes or more, two days or more a week, for over a year?	Yes or No
Eating speed	Is your eating speed faster than others?	Slow, Normal, or Fast
Frequent skipping breakfast	Do you skip breakfast three or more days a week?	Yes or No
Eating dinner late	Do you eat dinner within two hours before bedtime three days or more a week?	Yes or No
Frequent snacking	Do you eat snacks after dinner three days or more a week?	Yes or No
Alcohol drinking frequency	How often do you drink?	Rarely, Occasionally, or Everyday
Alcohol consumption	How much do you drink per day?	$<$1 glass of sake, 1–2 glass of sake, 2–3 glass of sake, or $\geq$3 glass of sake
Sleep habits	Do you sleep well and enough?	Yes or No

eTable 2. Sex and age distributions of respondents to the National Health and Nutrition Survey group[a]

Age group, years	Lifestyle survey		Physical condition survey	
Men				
40–49	553	(21.3)	382	(18.8)
50–59	519	(20.0)	378	(18.6)
60–69	713	(27.4)	589	(29.0)
≥70	814	(31.3)	683	(33.6)
Total	2,599	(100)	2,032	(100)
Women				
40–49	659	(21.4)	536	(21.1)
50–59	586	(19.0)	478	(18.8)
60–69	830	(26.9)	705	(27.8)
≥70	1,006	(32.7)	817	(32.2)
Total	3,081	(100)	2,536	(100)

Data are presented as number of subjects (%).

[a] Data were obtained from official reports of the 2015 National Health and Nutrition Survey.[5]
